# Effects of Toll-Like Receptors 3 and 4 in the Osteogenesis of Stem Cells

**DOI:** 10.1155/2014/917168

**Published:** 2014-12-25

**Authors:** Chen Qi, Xu Xiaofeng, Wang Xiaoguang

**Affiliations:** Department of Orthopedics, Affiliated Hospital of Jiangsu University, 438 Jiefang Road, Zhenjiang, Jiangsu 212000, China

## Abstract

*Objective*. To investigate the effects of Toll-like receptors in stem cell osteogenesis. *Methods*. Bone marrow mesenchymal stem cells (BMSCs) were divided into the blank group, the TLR-3 activated group, and the TLR-4 activated group. After 10 days' osteogenic-promoting culture, expression of type I collagen and osteocalcin was determined by Western blot. Osteoblasts (OBs) were also divided into three groups mentioned above. Alkaline phosphatase (ALP) and alizarin red staining were performed after 10 days' ossification-inducing culture. The expression of *β*-catenin was investigated by Western blot. *Results*. Both the TLR-3 and TLR-4 activated groups had increased expression of type I collagen and osteocalcin; the effect of TLR-4 was stronger. The intensity of alizarin red and ALP staining was strongest in the TLR-3 activated group and weakest in the TLR-4 activated group. Activation of TLR-4 decreased the expression of *β*-catenin, whilst activation of TLR-3 did not affect the expression of *β*-catenin. *Discussion*. This study suggested that both TLR-3 and -4 promoted differentiation of BMSCs to OBs. TLR-3 had an inducing effect on the ossification of OBs to osteocytes, whilst the effect of TLR-4 was the opposite because of its inhibitory effect on the Wnt signaling pathway.

## 1. Introduction

It is widely accepted that stem cells, as seed cells, in conjunction with bone tissue engineering scaffolding, are beneficial to bone healing due to their ability to differentiate to osteoblasts (OBs). Following ossification, OBs transform to osteocytes, which then form primary bone tissue. This is not, however, the final stage of bone formation. The physiological function of bone tissue does not develop until reconstruction of the bone tissue is completed by both OBs and osteoclasts (OCs). The whole process of the differentiation of stem cells to mature bone tissue is defined as osteogenesis of stem cells, which is regulated by various cellular factors, such as bone morphogenetic protein-2 (BMP-2) and fibroblast growth factor (FGF) [1, 2]. Toll-like receptors (TLRs) are one of these cellular factors that affect osteogenesis of stem cells [3, 4].

Toll-like receptors (TLRs) are important types of pattern-recognition receptors (PRRs) and are type I transmembrane glycoproteins. They are widely expressed in most human and other animal cells and tissues and not only initiate the primary immune response but also play an important role in the general immune system and the regulation of other bodily functions including proliferation of stem cells [5]. In addition, activation of TLRs can induce the secretion of various inflammatory factors such as interleukin (IL), tumor necrosis factor (TNF), and prostaglandin (PG), all of which have been shown to promote bone healing in the early stage of inflammation [6, 7]. It has been reported by Raicevic and his colleagues that TLRs are able to promote the osteogenic potential of human mesenchymal stem cells via inflammatory processes [8]. It is inferred that TLRs play important roles in the process of osteogenesis of stem cells. TLR-3 and TLR-4 are expressed on the surface of human bone marrow mesenchymal stem cells (BMSCs) [9] and have differing effects on the regulation of inflammation [10]. While TLR-3 promotes the secretion of interleukin-10 (IL-10), indoleamine 2,3-dioxygenase (IDO), and prostaglandin E-2 (PGE-2) and induces acute inflammation, TLR-4 promotes the secretion of IL-6, IL-8, and TNF-*β* and induces chronic inflammation. Consequently, it is reasonable to propose that the effects of TLR-3 and TLR-4 are most likely also different in the osteogenesis of stem cells.

The Wnt signaling pathway may be an important mechanism in the processes of osteogenesis of stem cells by regulating the OB function in gene transcription level [11, 12]. Activation of Wnt signaling pathway is able to enhance osteoblastogenesis [13] and promote bone formation [14]. *β*-catenin is the downstream signal molecule of Wnt pathway. Cytoplasmic *β*-catenin is accumulated as a result of the activation of Wnt signaling pathway and then transferred into cell nucleus. Nuclear *β*-catenin is able to upregulate the expression of RUX-2, which is crucial in the osteogenesis of stem cells, by integrating with T cell factor/lymphoid enhancer factor (TCF/LEF).

This study aims to determine the differing effects of TLR-3 and TLR-4 in the differentiation of BMSCs to OBs and the ossification of OBs to osteocytes. Another purpose is to investigate the effect of Wnt signaling pathway in the process of osteogenesis of stem cells.

## 2. Materials and Methods

Following isolation from rats (*n* = 5) as described [15], BMSCs were identified by flow cytometry. The BMSCs of 3rd generation were then divided into three groups: the TLR-3 activated group (*n* = 12 wells), the TLR-4 activated group (*n* = 12 wells), and the blank group (*n* = 12 wells). Cells in the TLR-3 activated group were treated with the TLR-3 agonist, polyinosinic-polycytidylic acid [Poly(I:C)] (SIGMA, USA, L2630-10MG), 10 ng/mL. Cells in the TLR-4 activated group were treated with lipopolysaccharide (LPS) (SIGMA, USA, L2630-10MG), 10 ng/mL, the activator of TLR-4. Cells in the blank group were not treated. All treatment groups were cultured in osteoblastogenic-promoting differentiating medium [10^−8^ mol/L dexamethasone, 10 mmol/L *β*-sodium glycerophosphate, 50 mg/L vitamin C, and H-DMEM with 10% fetal bovine serum (FBS)]. On day 10 of cell culture, the protein expression of type I collagen and osteocalcin of cells was detected by Western blot as previously described [16], in order to demonstrate the effects of TLR-3 and TLR-4 in differentiation of BMSCs to OBs. Quantitative analysis was also performed.

In order to demonstrate the effects of different TLR subtypes in ossification of OBs to osteocytes, alkaline phosphatase (ALP) and alizarin red staining were performed as following protocols. In the first 10 days, BMSCs isolated from rats were induced to OBs with osteoblastogenic-promoting differentiating medium mentioned above. OBs induced from BMSCs were divided into TLR-3 activated group (*n* = 12 wells), the TLR-4 activated group (*n* = 12 wells), and the bank group (*n* = 12 wells) and then treated with the same method mentioned above. Then, all cells in these groups were induced with ossification-inducing medium (3 mmol/L Na_3_PO_4_, 50 mg/L vitamin C, H-DMEM with 10% FBS) for another 10 days. After that, ALP staining and alizarin red staining were then performed to cells in these groups.

The OBs in the three groups mentioned above, TLR-3 activated group, TLR-4 activated group, and the blank group, were cultured for 10 days, and the expression of total *β*-catenin and nuclear *β*-catenin was detected by Western blotting.

## 3. Results

**Figure 1:**
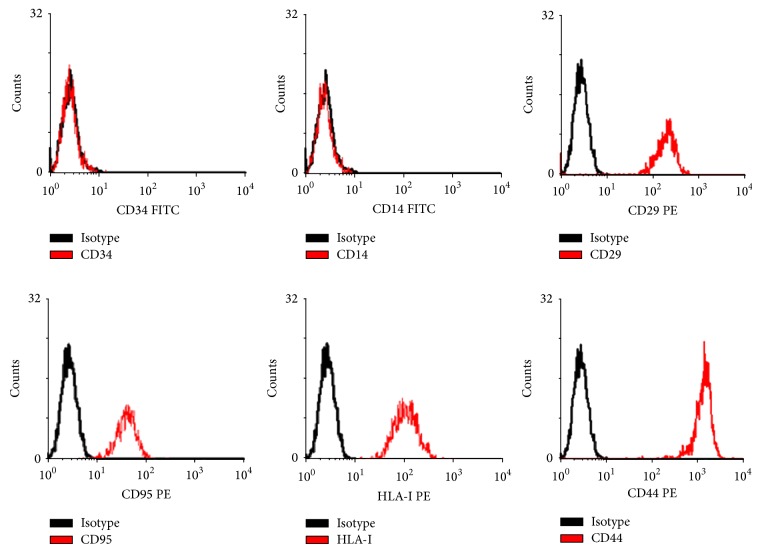
Identification of BMSCs by flow cytometry method. BMSCs were examined by flow cytometry for expression of discriminating BMSCs surface markers as described in Section 2. Shown in this set are representative findings for BMSCs: positive expression of CD29, CD95, HLA-I, and CD44, yet negative expression of CD34 and CD14 (*n* = 7). The black lines represent BMSCs incubated with corresponding isotype antibody controls.

**Figure 2:**
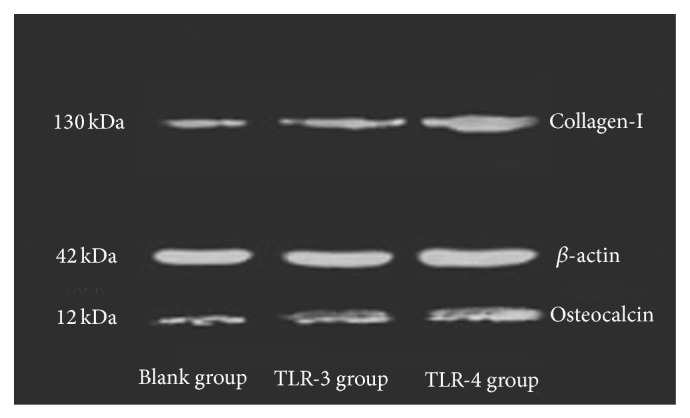
Western blotting of collagen-I and osteocalcin. BMSCs stimulation by different TLR ligands affected secretion of collagen-I and osteocalcin. The amount of collagen-I and osteocalcin was assessed by Western blotting analysis. Compared with BMSCs untreated which were used as negative control, cells stimulated by TLR-3 ligands and TLR-4 ligands secreted more collagen-I and osteocalcin (*n* = 12). The effect of TLR-4 ligands was stronger than TLR-3 ligands.

Results of flow cytometry showed that the surface markers of BMSCs were positive (Figure 1). The cells isolated from rats were identified as BMSCs, and following experiments were performed with these cells. In the present study, Western blot analysis (Figure 2) revealed that the expression of type I collagen was higher in the TLR-3 activated group (*t* = 9.258, *P* = 0.010 < 0.05) and the TLR-4 activated group (*t* = 23.469, *P* = 0.001 < 0.05) compared to the blank group. Moreover, cells in the TLR-4 activated group expressed higher levels of type I collagen than cells in the TLR-3 activated group (*t* = 9.061, *P* = 0.001 < 0.05). Similarly, the expression of osteocalcin was higher in the TLR-3 activated group (*t* = 5.854, *P* = 0.017 < 0.05) and the TLR-4 activated group (*t* = 20.663, *P* = 0.001 < 0.05) than that in the blank group. Between TLR-4 activated group and TLR-3 activated group, expression of osteocalcin was higher in the former one (*t* = 6.134, *P* = 0.004 < 0.05). Data of quantitative analysis of the western blot images was in Table 1.

**Figure 3:**
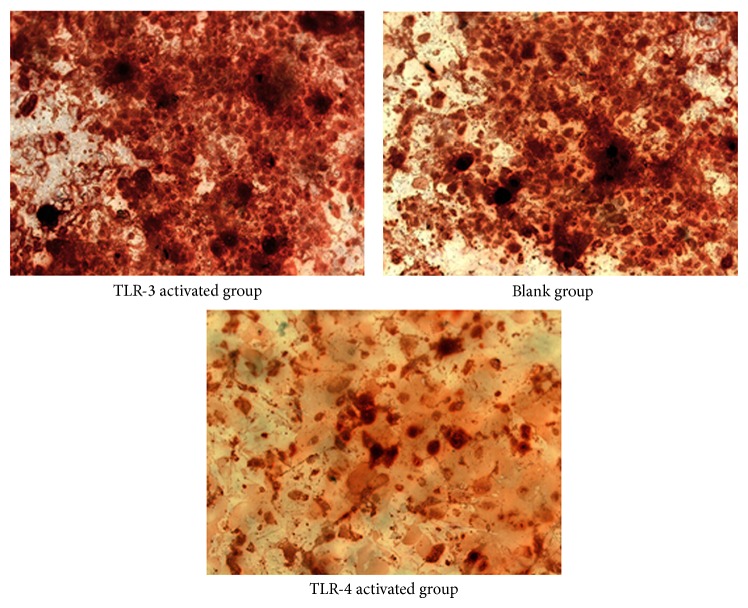


**Figure 4:**
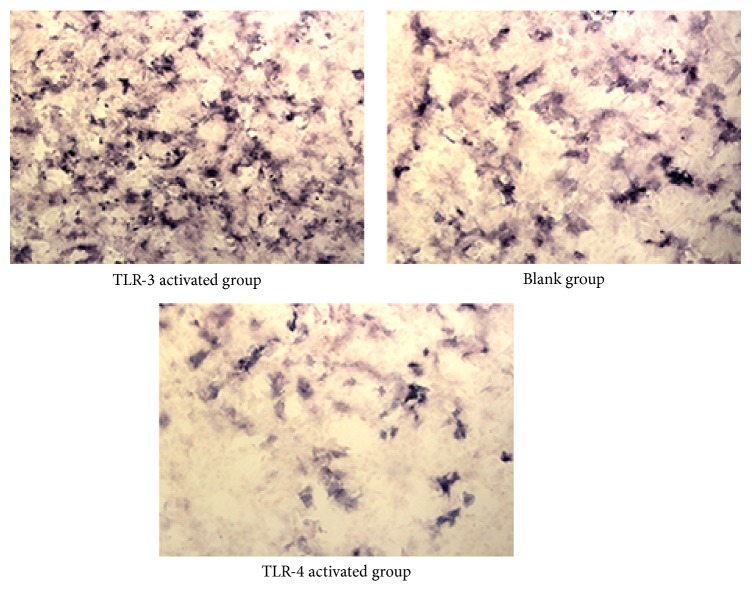


The intensity of alizarin red staining (Figure 3) in the blank group was stronger than that in the TLR-4 activated group (*t* = 4.017, *P* = 0.015 < 0.05) and even weaker in TLR-3 activated group (*t* = 4.791, *P* = 0.018 < 0.05). The result of ALP staining (Figure 4) by immunocytochemical (IHC) method was similar. The intensity of ALP staining was stronger in the TLR-3 activated group than that in the blank group (*t* = 3.811, *P* = 0.012 < 0.05), whilst the intensity of ALP in the blank group was stronger than that in the TLR-4 activated group (*t* = 4.115, *P* = 0.019 < 0.05). Data of quantitative analysis was in Table 2.

**Figure 5:**
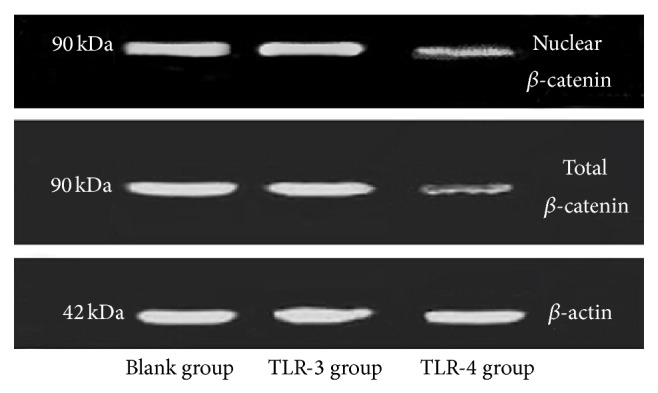
Expression of *β*-catenin. Western blotting analysis was used to access the expression of total *β*-catenin and nuclear *β*-catenin in OBs stimulated by TLR-3 ligands (*n* = 12) and TLR-4 ligands (*n* = 12). Untreated OBs were used as negative control (*n* = 12). More expression of *β*-catenin indicated better activation of Wnt signaling pathway. TLR-4 ligands inhibited Wnt signaling pathway, while TLR-3 ligands did not affect that mechanism.

When expression of total and nuclear *β*-catenin was mentioned (Figure 5), it was significantly lower in the TLR-4 activated group compared to the TLR-3 activated group, (*t*_total_ = 17.629, *P*_total_ = 0.001 < 0.05; *t*_nuclear_ = 16.177, *P*_nuclear_ = 0.003 < 0.05) and the blank group (*t*_total_ = 19.173, *P*_total_ = 0.001 < 0.05; *t*_nuclear_ = 17.992, *P*_nuclear_ = 0.005 < 0.05). However, there was no difference of the expression of *β*-catenin between TLR-3 activated group and the blank group (*t*_total_ = 1.727, *P*_total_ = 1.220 > 0.05; *t*_total_ = 1.675, *P*_total_ = 1.009 > 0.05). Data of quantitative analysis was in Table 1.

## 4. Discussions

This study clearly demonstrated that both TLR-3 and TLR-4 affected the osteogenesis of BMSCs. However, these two TLR subtypes played different roles in different stages of osteogenesis of BMSCs. The differentiation of BMSCs to OBs was promoted by the activation of both TLR-3 and TLR-4. Type I collagen and osteocalcin were detected to illuminate the effect of TLR-3 and TLR-4. Type I collagen, the primary composition of bone matrix and osteocalcin, is secreted by OBs. Secretion of type I collagen climaxes between day 10 and day 14, following the formation of OBs in preparation for the ossification to osteocytes [10]. In the last period of ossification, the levels of type I collagen decrease [17]. Osteocalcin, also secreted by OBs, is involved in an ossification negative feedback mechanism whereby secretion of osteocalcin inhibits overossification [18, 19]. The protein expression of type I collagen and protein expression of osteocalcin are indicators of viability of OBs. The results of the current study indicate that both TLR-3 and TLR-4 promote the differentiation of BMSCs to OBs. In addition, the activation of TLR-4 is more effective than the activation of TLR-3. The varying effects of TLR-3 and TLR-4 in stem cell osteogenesis may be attributed to the diverse inflammatory environment induced by the different TLR subtypes. In the acute inflammatory environment induced by overexpression of TLR-3, anti-inflammatory factors including IDO, PEG-2, and IL-10 are secreted whilst in chronic inflammatory environment induced by overexpression of TLR-4, proinflammatory factors such as TNF-*β*, IL-6, and IL-8 are secreted [10]. It has been reported that proinflammatory factors promote the differentiation of preosteoblasts [20]. Indeed a study by Glass et al. showed that TNF-*α*, a typical proinflammatory factor, augments the recruitment and differentiation of stem cells and as a consequence promotes bone healing [6]. Furthermore, fracture repair was delayed in IL-6 and TNF-*α* knockout mice compared to the wild types [21, 22]. Therefore it is possible that activation of TLR-4 may be involved in the promotion of proinflammatory factors associated with the differentiation of BMSCs to OBs.

During the ossification stage, activation of TLR-3 and TLR-4 showed opposite effects. ALP and alizarin red staining were performed to illuminate the effect of ossification. Mature OBs release a large number of matrix vesicles, each of which contains a large amount of calcium and is helpful in mineralization [23, 24]. Alizarin red stains these calcium stores orange, and these calcium stores can then be microscopically analyzed. It has been reported that ALP is expressed in the membrane of matrix vesicles released by mature OBs. Consequently, ALP is regarded to be one of the primary regulators of the ossification of OB [25]. In OBs with tissue nonspecific alkaline phosphatase negative (TNAP−/−), formation of bone nodule and ossification cannot be observed; however, OBs with TNAP+/− can form bone nodule, while ossification was delayed [25]. Alizarin red staining and ALP staining are therefore the most direct and efficient methods to determine the ossification of OBs. The results demonstrated that while ossification of OBs to osteocytes was enhanced by TLR-3, it was inhibited by the activation of TLR-4.

Given that activation of both TLR-3 and TLR-4 had the same effect on the promotion of the differentiation of stem cells to OBs, it was interesting to observe that TLR-3 and TLR-4 had differing effects on the ossification of OBs to osteocytes. It became clear that it was necessary to investigate the downstream mechanisms of TLR-3 and TLR-4, as alterations in these downstream mechanisms may provide insight into the differing effects of TLR-3 and TLR-4 in stem cell osteogenesis. The Wnt signaling pathway promotes bone formation and inhibits bone absorption and is one of the most important signaling pathways of bone metabolism [26]. Existence of structurally stable and soluble *β*-catenin in the cytoplasm, the downstream signaling molecule of the Wnt signaling pathway, is vital for complete transduction of the Wnt signaling pathway [27]. Lack of *β*-catenin results in abnormal bone metabolism and dysfunction of bone [26]. It has been reported that TLR-4 inhibits the Wnt signaling pathway in intestinal epithelial cells of new born mice [28]. Consequently, it is possible that TLR-4 may also inhibit the Wnt signaling pathway in OBs in rats. To investigate this possibility, the expression of *β*-catenin in OBs was investigated in the TLR-4 activated group and also in the TLR-3 activated group. The results in current study support the inhibitory effect of TLR-4 in the Wnt signaling pathway, thereby possibly explaining the inhibitory effect of TLR-4 in the ossification of OBs to osteocytes, observed in the present study. However, activation of TLR-3 did not affect the expression of *β*-catenin and the Wnt signaling pathway. Therefore, TLR-3 shows differential effect in ossification of OBs compared to TLR-4.

## Figures and Tables

**Table 1:** Quantitative analysis of Western blotting ($\bar{x}\;\pm \;s$).

	Collagen-I	Osteocalcin	Total *β*-catenin	Nuclear *β*-catenin
Control group	0.063 ± 0.004	0.322 ± 0.018	0.806 ± 0.022	0.792 ± 0.031
TLR-3 group	0.209 ± 0.027	0.509 ± 0.052	0.788 ± 0.019	0.769 ± 0.017
TLR-4 group	0.401 ± 0.025	0.719 ± 0.028	0.116 ± 0.030	0.181 ± 0.028

**Table 2:** Quantitative analysis of alizarin red and ALP staining ($\bar{x}$  ±  *s*).

	Alizarin red staining	ALP staining
Control group	0.277 ± 0.016	0.401 ± 0.020
TLR-3 group	0.511 ± 0.035	0.657 ± 0.032
TLR-4 group	0.082 ± 0.020	0.109 ± 0.011
